# Plain radiography in patients treated with intrathecal drug delivery using an implantable pump device

**DOI:** 10.1007/s13244-017-0568-z

**Published:** 2017-08-24

**Authors:** Elmar M. Delhaas, Biswadjiet S. Harhangi, Sander P. G. Frankema, Frank J. P. M. Huygen, Aad van der Lugt

**Affiliations:** 1 Center for Pain Medicine, Erasmus MC University Medical Center, ‘s-Gravendijkwal 230, 3015 CE Rotterdam, The Netherlands; 2 Department of Neurosurgery, Erasmus MC University Medical Center, Rotterdam, The Netherlands; 3 Department of Radiology, Erasmus MC University Medical Center, Rotterdam, The Netherlands

**Keywords:** Radiography, Diagnostic imaging, Spinal infusions, Implantable infusion pumps, Adverse events

## Abstract

**Objectives:**

Intrathecal drug administration using an implanted pump system is well established in intractable spasticity and pain. However, despite continuous advancements in manufacturing technology, adverse events related to the pump and catheter still occur. Most of them, such as migration, damage, disconnection and occlusion, are related to the spinal catheter. The aim of this overview is to update radiologists on how plain radiography of the implanted delivery system for intrathecal drug administration should be interpreted and to increase awareness for the need of urgent and timely multidisciplinary troubleshooting.

**Methods:**

Plain radiographic images of patients treated with intrathecal drug administration using an implantable drug delivery system were analysed in a multidisciplinary setting at our (university) referral centre for complications in intrathecal drug administration.

**Results:**

Examples of catheter-related adverse events are described and a proposal is made for stepwise interpretation of standard plain radiographic images.

**Conclusions:**

Plain radiological images are the mainstay for the diagnosis of catheter-related adverse events in intrathecal drug delivery. Radiologists play an important role in an early diagnosis. An awareness of abnormal radiological findings seems important to avoid a life-threatening withdrawal syndrome.

**Teaching points:**

• *Untimely cessation of intrathecal drug delivery can lead to a life-threatening withdrawal syndrome.*

• *Initially mild symptoms can lead to an exacerbation of a withdrawal syndrome.*

• *Most intrathecal catheter-related problems are visible on plain radiography.*

• *Common causes of catheter problems are migration, lacerations, occlusion and disconnection.*

• *Knowledge on implanted intrathecal catheters is crucial for interpretation of plain radiography.*

## Introduction

For over 30 years intrathecal drug delivery systems have been successfully applied in thousands of patients for the management of spasticity [1, 2] and dystonia (both intrathecal baclofen) [3], and for chronic pain (intrathecal analgesic drugs) [4, 5]. Compared with oral administration, infusion directly into the cerebrospinal fluid (CSF) has an extended treatment effect and with fewer unwanted side effects [1, 6]. There is general consensus that intrathecal therapy should be reserved for patients who have an insufficient response to more conservative therapies and/or for patients who experience serious side effects [7].

Despite generally favourable and safe outcomes [8, 9] and continuous advancements in manufacturing technology, pump and catheter-related adverse events still occur [10]. Although the benefits usually outweigh the risks [11], even limited exposure to adverse events remains a problem. Early recognition of complications and their prompt management is needed.

Most of the drug delivery device-related adverse events are caused by intrathecal catheter failure [12–14]. Approximately 15–40% of patients experienced catheter complications [2, 15–18]. With the newly developed Ascenda catheter instead of the older silicone catheters, a tremendous reduction from 18 to 1.1% was reported by Motta and Antonello [19]. The main reasons for this are migration, lacerations, occlusion and disconnection of the catheter, which cause a sudden cessation of intrathecal drug administration. Abrupt interruption of intrathecal baclofen delivery can present within several to 48 h with a spectrum of signs and symptoms. Then, the initially mild symptoms of exacerbation of spasticity, fever, excessive sweating and pruritus can escalate to a life-threatening multi-organ failure. Over time, hyperthermia develops with values up to 42° C [20], accompanied by nausea, respiratory distress, hypotension, tachycardia, hallucinations, delirium, disorientation, psychosis, sometimes with seizures, rhabdomyolysis with increased creatinine kinase levels resulting in disseminated intravascular coagulation and multi-organ failure [13, 21–29]. In rare cases, intrathecal baclofen withdrawal can even be fatal [30, 31]. The symptoms are probably related to the release of excitatory neurotransmitters that occurs when baclofen-mediated inhibition GABA-B effect is abruptly interrupted [32–34]. The heterogeneous symptoms occurring during withdrawal of intrathecal baclofen and of intrathecal opioid treatment may result in misdiagnosis, wrong referrals and (eventually) in a disastrous treatment delay. An additional problem is the referral of the patient in good time to a specialised centre, which is a requisite for successful treatment [33]. Different imaging techniques, including plain radiography, fluoroscopy with contrast material injection via the access port of the pump, CT myelography, MRI and ^111^In-DTPA scintigraphy, are used to diagnose malfunction of the drug delivery system [14, 36–41]. Of all these imaging modalities, plain radiography is the most important, especially in an acute situation. Furthermore, a rigorous and adequate interpretation of the images by the radiologist is crucial to make a correct diagnosis and, if necessary, implement (urgent) interventions.

This overview aims to offer the radiologist a systematic approach for the evaluation of all parts of the intrathecal delivery system on plain radiography. This may help radiologists to identify causes of drug delivery failures in emergency and in chronic situations.

## Materials and methods

Below, we describe the most commonly used methods: (1) intrathecal drug delivery system, (2) intrathecal catheter and (3) surgical implantation technique.

### Intrathecal drug delivery system

**Fig. 1:**
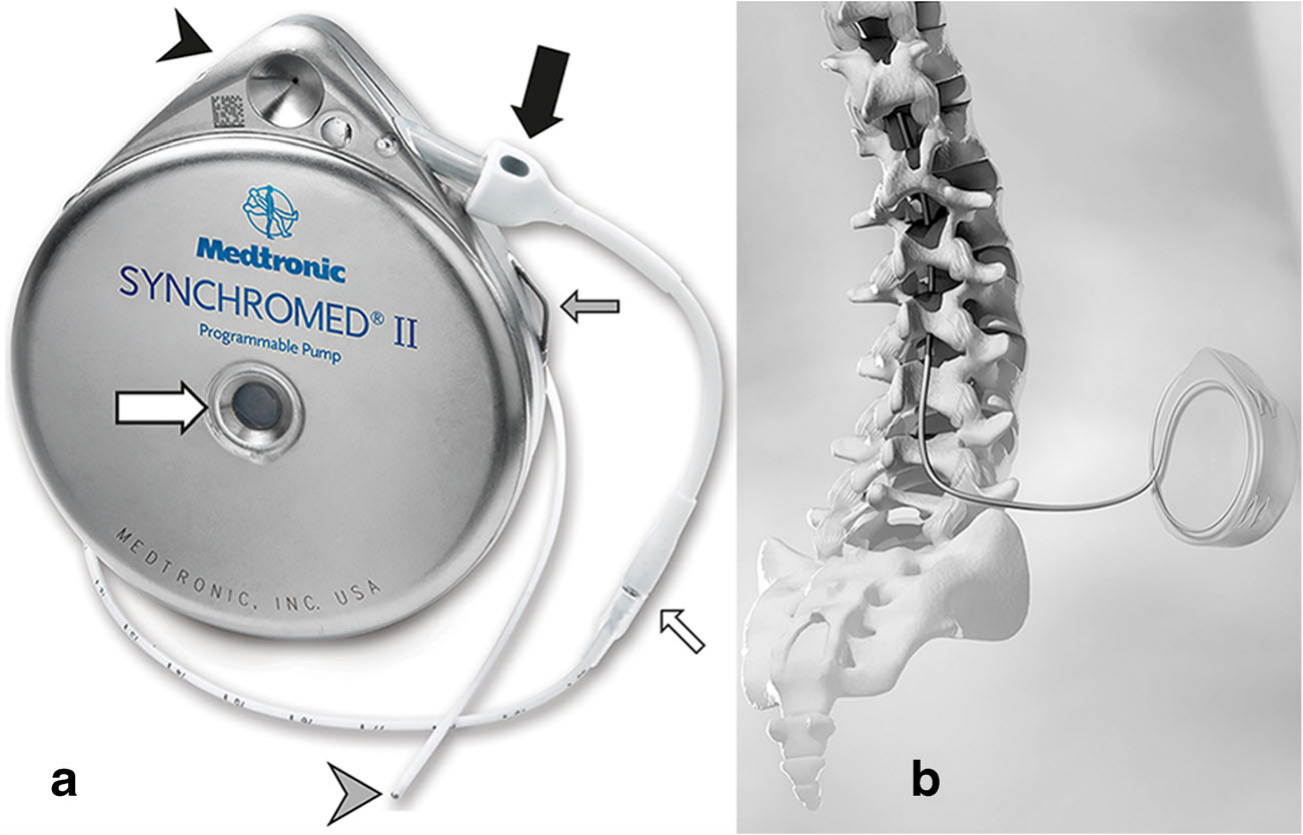
Implanted, programmable pump system. External view (**a**) and drawing of implanted pump and intrathecal catheter 8731SC (**b**). Pump with the catheter access port (*black arrowhead*), pump catheter connection (*thick black arrow*), refill membrane (*thick white arrow*) and suture loops for fixation (*thin grey arrow*), catheter-catheter segment connection (*thin white arrow*) and titanium catheter end (*grey arrowhead*)

Although several pump systems are available, the implantable Synchromed II pump (Medtronic, Minneapolis, MN, USA) is by far the most rigorously tested and most applied implanted programmable device, worldwide (Fig. 1). Here, we focus on this pump only.

The implantable Synchromed II pump and the related intrathecal catheters have received the Conformité Européenne (CE) mark and are approved by the U.S. Food and Drug Administration (FDA) for treatment of pain and spasticity. During continuous intrathecal drug delivery, the prescribed medication is administered through an intrathecal catheter, connected with an implantable programmable pump system. The pump provides precise intrathecal drug delivery to patients with spasticity or chronic intractable pain. Via the refill septum, which is in the centre of the device (Fig. 1a), the reservoir is filled percutaneously. The gas below the reservoir exerts pressure which advances the drug into the inner tubing of the pump. The accompanying programmer device enables the delivery rate and mode to be programmed. A rotor system pushes the programmed dose with precision through the catheter access port via the catheter into the intrathecal space.

### Intrathecal catheter

Over time, several catheter types for the implantable Synchromed II pump have been developed and are commercially available. Although two types of intrathecal catheters (the 8731SC, and the Ascenda) are currently available, older types are still in use. Determining the type of implanted catheter via plain radiography is important for correct interpretation.

**Fig. 2:**
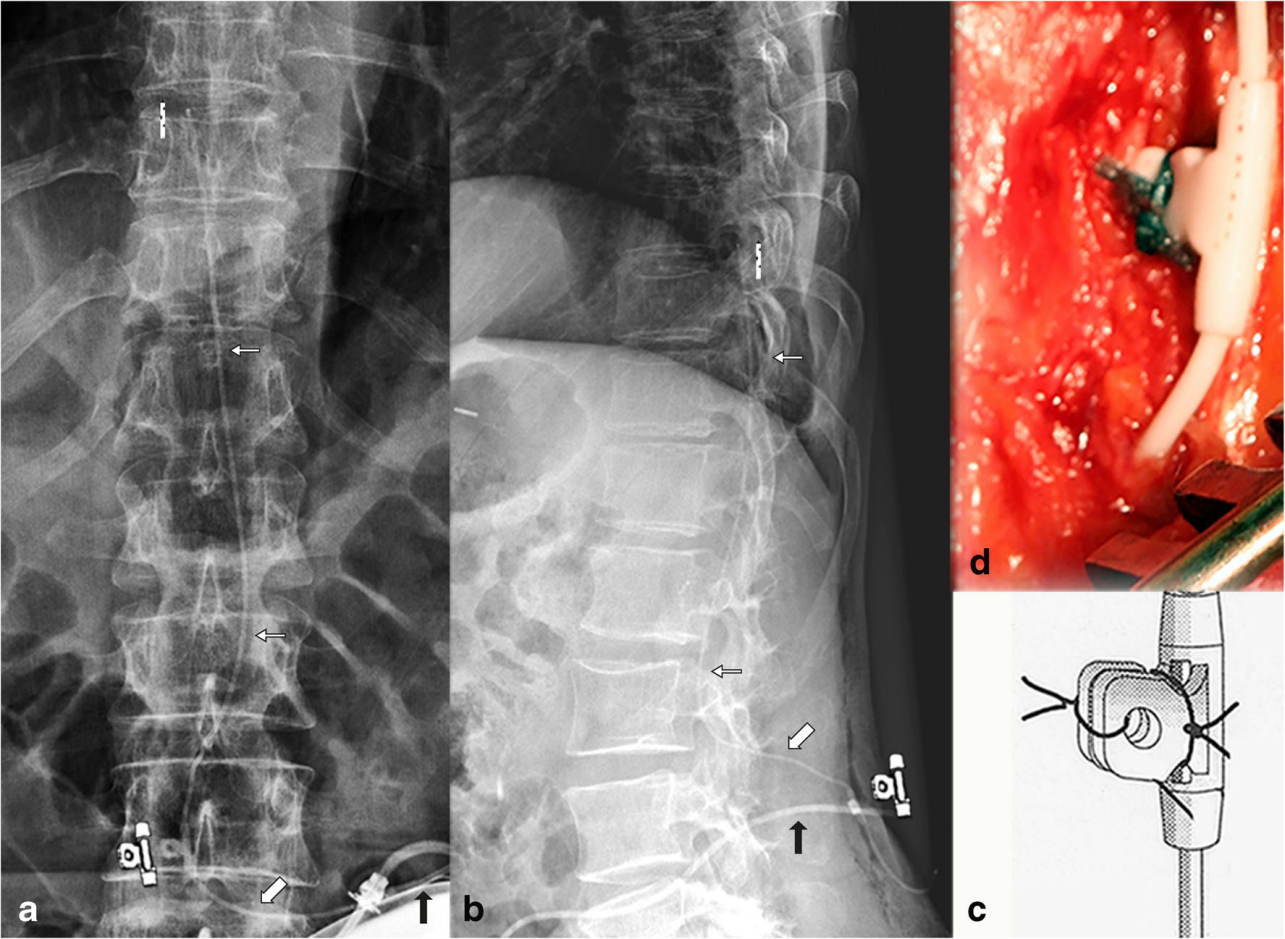
Anterior-posterior (**a**) and lateral (**b**) plain radiography of the lumbar spine, in vivo image (**d**) and artist rendering (**c**) of the typical *folded* fixation anchor (with *anchor symbol*), large diameter catheter pump segment (*black arrow*), small diameter outside spinal canal (*thick white arrow*) and intrathecal (*small white arrow*, *catheter end symbol*) segment of the 8731SC catheter

The 8731SC catheter is normally visible on plain radiography (Fig. 2; *see* Table 1 for a key to the symbols used in the figures). The pump-catheter connection is sutureless and the two segments of the catheter have a different diameter, i.e. the pump segment of the catheter has a larger outer diameter than the spinal segment. Furthermore, the 8731SC catheter is provided with a two-pin catheter-catheter connector, a folded V-wing anchor which is fixed on the fascia, and has a catheter-end with six side holes and a round titanium tip. The folded V-wing in the 8731SC catheter locks the catheter in place (Fig. 2c, d) and is, therefore, important for the diagnosis of catheter disorders. A partially or completely unfolded anchor (*see* Fig. 9) creates a major risk for dislodgement, potentially leading to a complete migration.

**Table 1:**
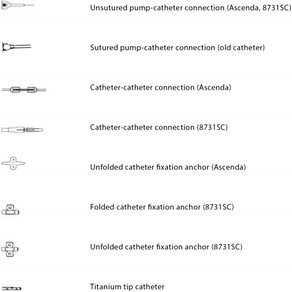
Explanation of the symbols

**Fig. 3:**
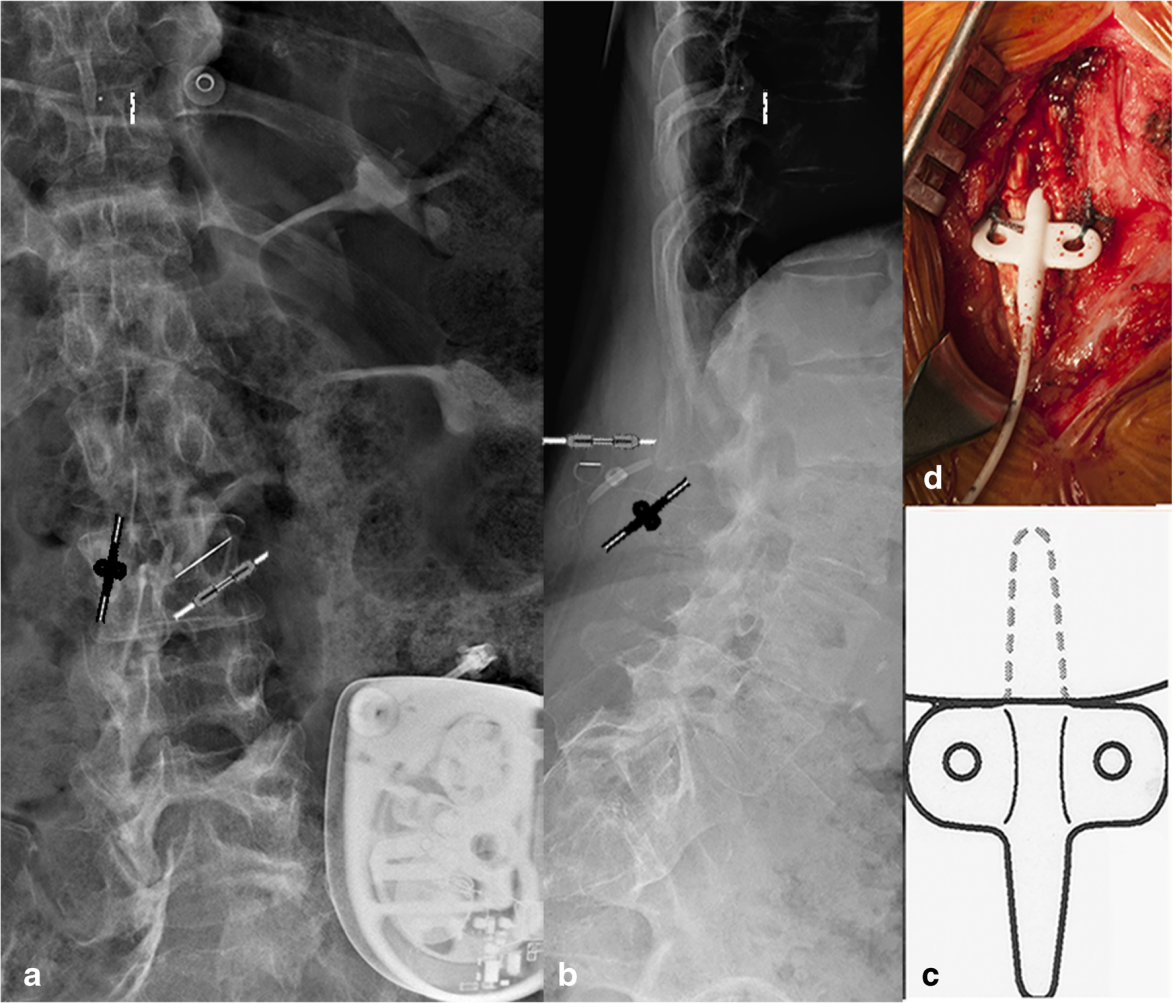
Anterior-posterior (a) and lateral (**b**) plain radiography of the lumbar spine, in vivo image (**d**) and artist rendering (**c**) of the typical *unfolded* fixation anchor of the Ascenda catheter. The small diameter catheter pump is hardly visible. The only reference points (**a, b**) are the needle connector (*needle symbol*), the unfolded anchor (*anchor symbol*), and the titanium catheter tip (*catheter end symbol)*

The Ascenda catheter (Fig. 3) has a poor opacity and the pump-catheter connection is also sutureless. Both catheter segments have the same diameter and are connected to each other with a needle connector. The fixation on the fascia is conducted by an unfolded V-wing anchor (Fig. 3c, d). This catheter also has a catheter-end with six side holes and a round titanium tip. In contrast to the 8731SC catheter, the unfolded V-wing in the Ascenda catheter locks the catheter in place.

Older catheters have a sutured pump-catheter connection and distinct differences in anchoring (this could even be absent), different catheter-catheter connection, different catheter ends, and the presence or absence of a mushroom-shaped titanium tip. Either a so-called ‘one’ piece catheter type with only a connection at the pump or a two-segment catheter are used.

### Surgical implantation technique drug delivery system

Implantation is performed under local anaesthesia with intravenous sedation or general anaesthesia, With the patient in lateral decubitus position, a 3– to 4-cm dorsal midline incision is made at the planned implantation level up to the muscular fascia. On level L2-L3 or L1-L2 a silicone catheter is obliquely inserted into the intrathecal space, using a 15-G (8731SC catheter) or 16-G (Ascenda catheter) introduction (Tuohy) needle. The introduction needle is inserted 1–2 cm using a paramedian approach [42] to prevent catheter shearing and crushing by the frequent movements of the vertebral spinal process when a midline approach is applied. Under fluoroscopy the catheter is advanced to the mid-thoracic level [43–45]. Some physicians claim that positioning of the tip at the high thoracic, or even cervical position, gives a better result on the upper extremities; however, this has not yet been proved [46, 47]. At the abdominal site, a subcutaneous pocket is made in which the pump is placed and sutured using the outside suture loops of the pump. The pump catheter segment is connected suturelessly to the pump and tunnelled from the pump pocket to the dorsal incision, where it is connected to the spinal catheter part. The excess catheter length should be placed dorsal to the pump in the pocket.

### Radiological examination after implantation of an intrathecal drug delivery system

Since the quality of the perioperative fluoroscopic images is insufficient for detailed information, routine postoperative plain radiography is performed after all of the surgical procedures and manipulations. For adequate interpretation, plain radiography of the pump and the entire implanted catheter in two directions is required [36].

### Development of a stepwise interpretation schedule

A stepwise interpretation of the standard plain radiographic images has been developed (Appendix) to offer the radiologist a systematic approach for the evaluation of all parts of the intrathecal delivery system on plain radiography. This stepwise interpretation is based on expert opinion.

## Results

The normal and abnormal plain radiographic findings are described in a 14-step approach, a pump roller examination is also described.

### Fourteen-step interpretation of standard plain radiographic images

Step 1 Previous radiographs available?

Comparison with previous radiological examinations is required to detect subtle or overt changes in the location and position of the pump and catheter.

Step 2 Type of catheter used.

Primarily the use of a 8731SC catheter (Figs. 1a, 2, 5, 9, 10, 11), an Ascenda catheter (Figs. 3 and 6) or an older one (Figs. 4 and 8) should be determined.

**Fig. 4:**
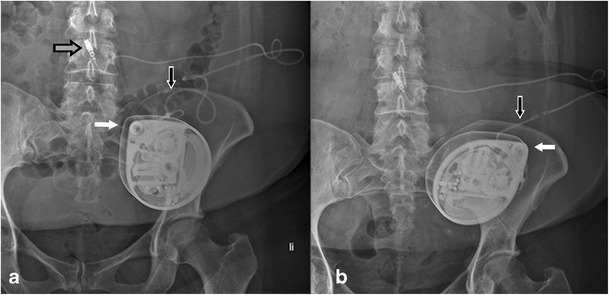
Reel syndrome with signs of withdrawal syndrome in a 52-year-old woman with dystonia in CRPS. The apex of the pump is turned from the 11 o’clock to 2 o’clock position (*white arrow*). Because of the visible traction on the catheter (*black arrow*), the pump must be rotated several times about the horizontal axis

**Fig. 5:**
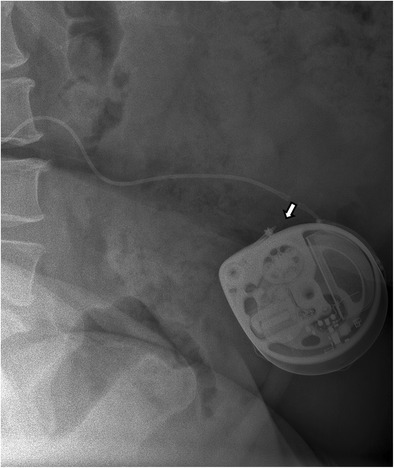
Disconnection at the pump-catheter site (*white arrow*) with signs of withdrawal syndrome in a 52-year-old woman with dystonia in *complex regional pain syndrome*

**Fig. 6:**
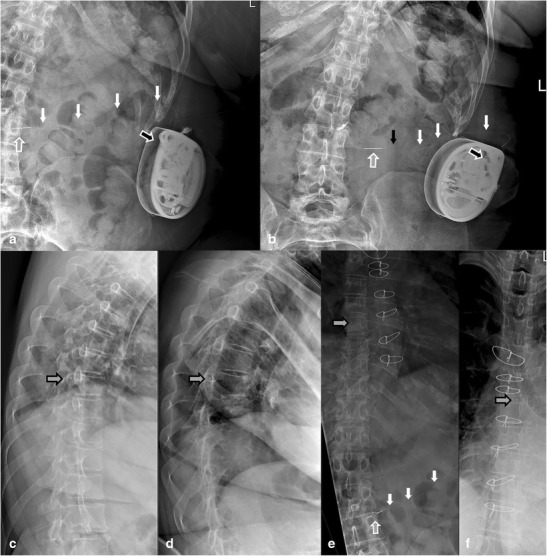
Reel syndrome with signs of withdrawal syndrome in a 45-year-old extremely obese woman with spasticity in multiple sclerosis. Baseline plain radiography (**a, c, e**) with follow-up images after 4 weeks (**b, d, f**). The apex of the pump is turned from the 11 o’clock (**a**) to the 2 o’clock (**b**) position (*black-white arrow*). The Ascenda needle connector is moved from the original position (**a**) to a more lateral position (**b**) (*grey-white arrow*) with disconnection of catheter pump segment (*black arrow*). The Ascenda catheter is hardly visible (white arrows). The tip of the catheter is unchanged (**c–f)** at level T8 (*grey-black arrow)*

**Fig. 7:**
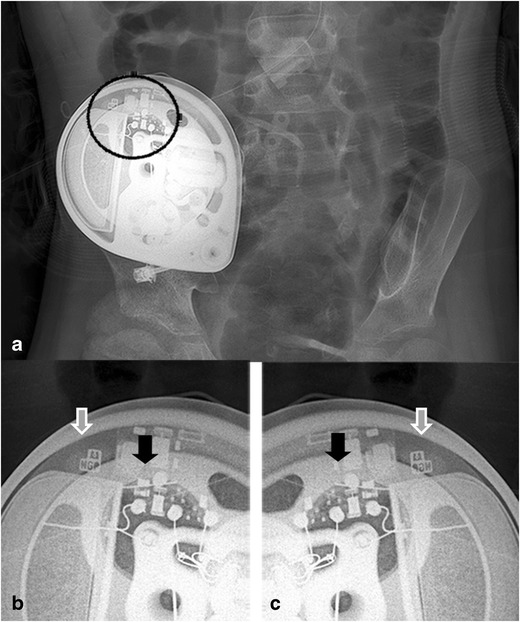
A magnification view of the *black circle* (**a**) shows the radiopaque pump identifier consisting of the logo of the manufacturer and three alphabetic letters (*white arrow*) with visible electronics (**b**) into the left-hand side (*black arrow*). For (some) odd rotations of the pump to the vertical axis (Twiddler’s syndrome), the pump is located on the head, which can be recognised by the logo in mirror image, and the electronics to the right-hand side (**c**)

**Fig. 8:**
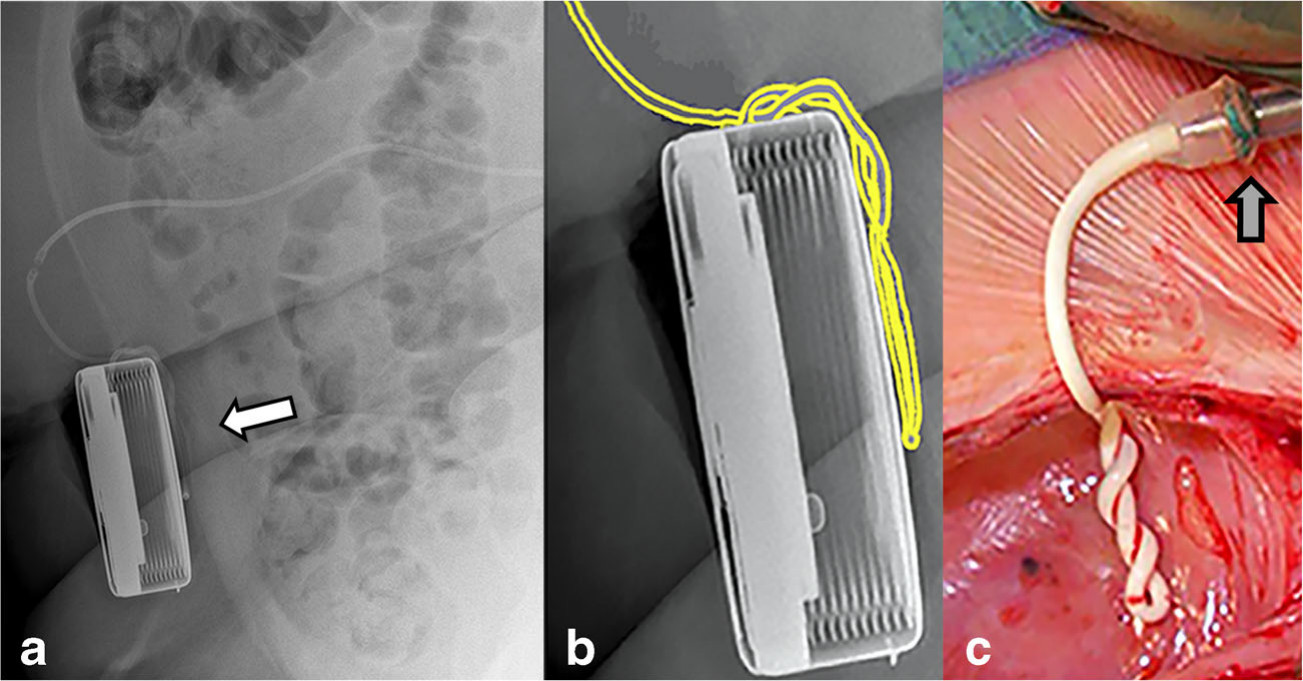
Twiddler’s syndrome with signs of withdrawal syndrome in a 51-year-old woman with dystonia in complex regional pain syndrome. A repeatedly twisted old type catheter is visible on lateral plain radiography behind the implanted pump (*white arrow*) (**a**), magnification of the pump with artist rendering (**b**) and an in vivo image (**c**). The catheter is fixed to the pump with an old sutured connector (*grey arrow)*

**Fig. 9:**
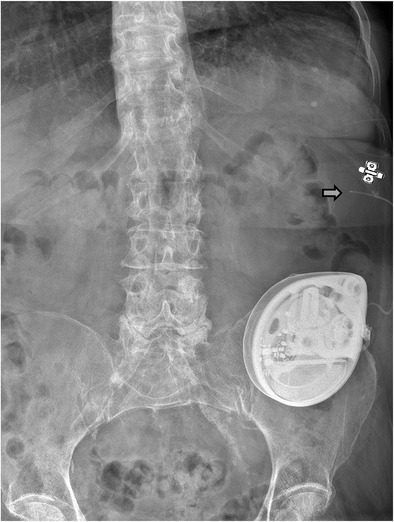
Catheter 8731SC migration (*gray arrow*) caused by an open V-wing anchor (*anchor symbol*) in an 80-year-old woman with intractable pain and spasticity caused by failed back surgery syndrome, with a spinal cord lesion (SCL) T12 treated with intrathecal baclofen

**Fig. 10:**
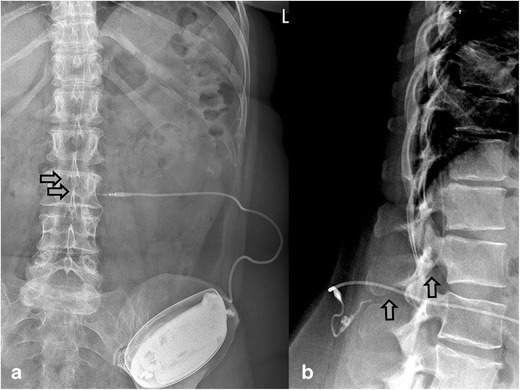
Sheared catheter 8731SC (*gray arrow*) with retracted segment partially outside the spinal canal, with no cerebrospinal leakage and no clinical signs of postural spinal headache or an intracranial hypotension syndrome, in a 48-year-old woman with cerebral palsy treated with intrathecal baclofen

**Fig. 11:**
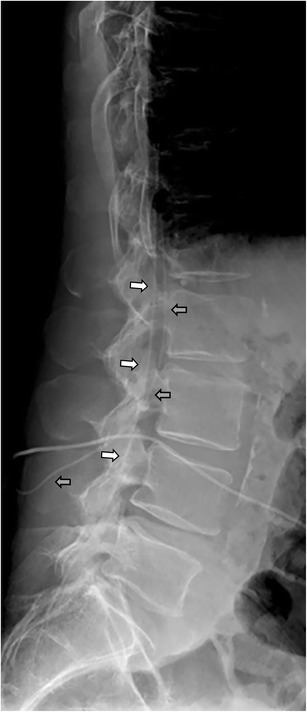
A 62-year-old man with treatment-resistant spasticity was successfully treated for many years with intrathecal baclofen. During exacerbation of the clinical symptomatology, the catheter 8731SC was found to be torn off and left behind in the spinal canal (white arrow). For further treatment, a new catheter, of the same type was inserted (*gray arrow*)

Step 3 Pump position

**Fig. 12:**
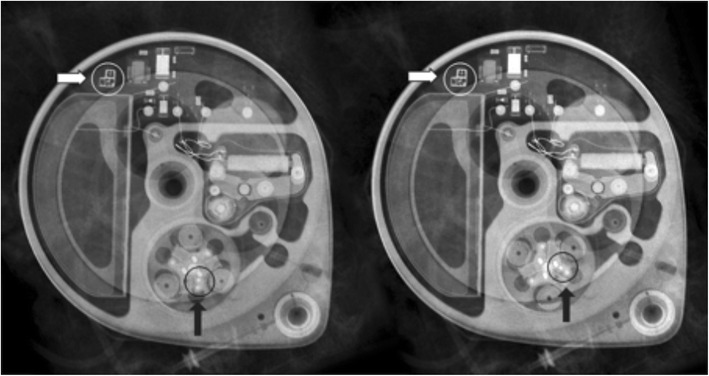
In this pump rotor examination, a normal rotation was found (change in position indicated by the *black arrows*). The *white arrows* show the pump position identifier (Fig. 7)

The position of the pump is described by reporting the pump apex (Figs. 1a, 3, 4, 5, 6, 7, 9, 10, and 12) as a location on the clock face and the abdominal quadrant in which the pump is placed. To exclude pump rotations, it is important to compare the current pump position with previous radiological images. Rotations about the horizontal axis are known as the Reel syndrome [48, 49] (Figs. 4 and 6), resulting in traction on the catheter and risk of disconnection at the level of the pump-catheter (Fig. 5) or catheter-catheter connection (Fig. 6b).

Step 4 Pump-catheter connection

On plain radiography, the pump-catheter connection is clearly visible (Figs. 2, 3, 4, 5, and 9).

Step 5 Excess pump catheter segment behind the pump

In common practice, the excess catheter length is positioned behind the pump. In case the catheter surplus is located above the pump reservoir, refilling with medication or injecting contrast material in the catheter access port might result in catheter puncturing. Catheter perforation can result in dose adjustment problems, or even in a severe withdrawal syndrome [38].

Step 6 Control identifier

With the magnification view the radiopaque identifier with the company logo and three alphabetic letters can be recognised opposite the pump apex (Fig. 7). When the pump is rotated around its longitudinal axis an odd number of times, the identifier is visible as a mirror image. This rotation was originally described in cardiac pacemakers [50] and is known as Twiddler’s syndrome. The rotation can occur either spontaneously or as result of repeated twiddling with the device by the patient. The syndrome has also been described in an implanted pump for intrathecal drug administration [51]. After several rotations (Fig. 10), the catheter will occlude, rupture or dislodge resulting in termination of the drug delivery.

Step 7 Catheter pump segment

The 8731SC thick-walled pump catheter segment is clearly visible (Figs. 1a, 2, 5 and 10) and in most cases the spinal segment is also visible (Figs. 2, 9, 10 and 11) The lack of opacity of the Ascenda catheter creates problems with visualisation of the pump segment (Figs. 5 and 6). Normally, this segment is clearly visible in older catheters.

Step 8 Catheter-catheter connector

The standard position of the clearly visible catheter-catheter connector is near the spine (Figs. 3, 4, 6b and 10). However, if the connection is placed in the pump pocket, recognition can be difficult. In the 8731SC catheter, the connector is visible as a two-pin connector (Fig. 10), in the Ascenda catheter as a needle (Figs 3 and 8a,b,e), and in older catheters often as a sharp small two-pin connector.

Step 9 Spinal segment of the catheter outside the spinal canal

The spinal segment of the 8731SC catheter has a smaller diameter than the pump segment and seems to be vulnerable to twisting, migration (Fig. 9) and shearing (Fig. 10). The pump segment and the spinal segment of the Ascenda catheter have the same small diameter. The improved mechanical properties make it less vulnerable. Kinking cannot be demonstrated on plain radiography in all implanted catheters. For a definite conclusion, additional investigations, such as injection of contrast material or scintigraphy, are needed.

Step 10 Different anchors

A folded anchor in the 8731SC catheter (Fig. 2) and an unfolded anchor in the Ascenda catheter (Fig. 3) are present. In older catheters, both folded and unfolded anchors can be found. At the anterior-posterior view, anchors are often poorly visible (Fig. 3a); whereas on the lateral image they are adequately visible (Fig. 3b).

Step 11 Catheter insertion, midline or paramedian?

Inserting an intrathecal catheter using the midline approach raises the risk of catheter crushing due to spinal column movements (Fig. 10). Therefore, a paramedian approach is the standard implantation technique.

Steps 12–13 Spinal intrathecal catheter segment

Due to the poor visibility of the Ascenda catheter, the thoracic or the cervical vertebral column catheter segment is inadequately imaged. This leads to lack of information on the position of the catheter.

Due to the titanium end, the radiopaque tip can be recognised (Figs. 3 and 6). Attention must be paid to retained catheter fragments (Fig. 11). This can occur at the time of catheter insertion, removal or as a late complication [52, 53]. There is no consensus about the treatment of retained fragments. Both conservative [52, 53] (Fig. 11) and surgical treatment [52, 54] are performed. Nevertheless, serious complications like subarachnoid haemorrhage [39, 55, 56] and migration into the ventricle [56] are reported. Special attention must be paid if an intrathecal fragment also remains partially outside the spinal canal. Leakage of CSF with the development of postural headache, a pseudomeningocele [56] or even an intracranial hypotension syndrome could occur. However, sometimes no CSF leakage is present (Fig. 10). In a conservative approach, the position of the retained catheter fragment should be followed-up over time.

Step 14 Radio-opaque catheter tip

In the latest catheters, a titanium tip has been built into the end of the catheter. In these intrathecal catheters, on plain radiography this tip is recognised as a ball at the end of the catheter (Figs. 2, 3 and 6).

### Special pump roller examination

If the rotor of the pump stalls, a two-tone emergency alarm will sound. After interrogation with the device programmer, the device display indicates a ‘Motor Stall’. When no motor stall is indicated and this is not derived from the pump logs, a roller study should be performed in case failure is suspected (Fig. 12). In this procedure, the pump is programmed in the continuous infusion mode, using a preset, without activation and with a priming bolus of 10 μl with a duration of 1 min. With fluoroscopy, the rotor is visualised, thereby reducing the aperture. A plain radiographic image is made and the preset bolus delivery is activated. After 2 min, a new plain radiographic image is made and the two images are compared. In a normal pump function, the rollers have moved approximately 60° from their original position. The extra radiopaque dot on one of the roller arms helps to visualise the roller movement (Fig. 12).

## Discussion

Although intrathecal drug administration using an implanted pump system has been employed for many years in therapy-resistant spasticity and intractable pain, knowledge on diagnostic imaging during adverse events remains limited.

The main reasons for this limited knowledge include (1) the diminished frequency of catheter-related treatment failures due to advancements in manufacturing and (2) the limited application of the treatment in different clinics. While plain radiography is the mainstay for the diagnosis of drug delivery device-related adverse events, radiologists play an important role in early diagnosis. An awareness of typical radiographic images in relation to intrathecal catheter failure is important to avoid a (sometimes life-threatening) withdrawal syndrome. The most frequent causes of drug delivery failure are migration, damage, disconnection and occlusion of the spinal catheter. In our opinion, applying the presented 14-step analysis and increasing the awareness of abnormal radiological findings will help physicians to avoid a life-threatening withdrawal syndrome.

A limitation of this proposal is that our approach has not yet been validated but is based on our expert opinion. Due to the present lack of high-quality evidence, we strongly believe that, as a first step in quality improvement, the current variation in radiological practice should be avoided. The stepwise approach, as proposed by our group, might be an effective first step towards raising the quality of care related to troubleshooting for intrathecal drug delivery with implanted systems.

## Figures and Tables

**Table 2:**
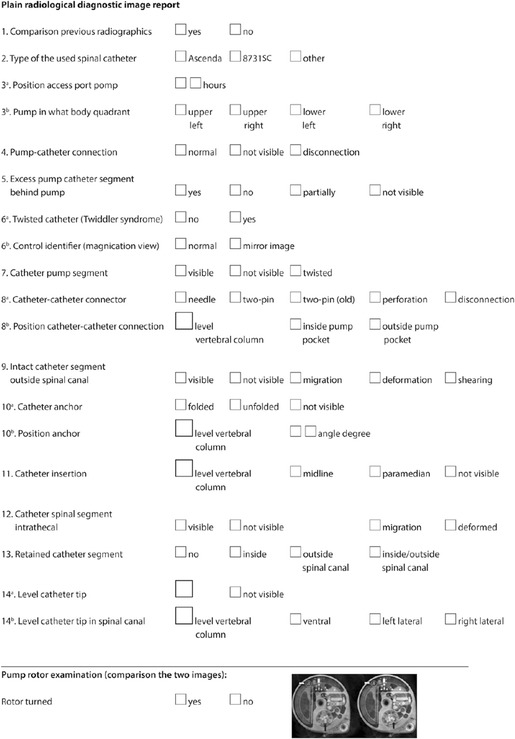
Plain radiography image report
